# CBT reduces CBF: cognitive‐behavioral therapy reduces cerebral blood flow in fear‐relevant brain regions in spider phobia

**DOI:** 10.1002/brb3.510

**Published:** 2016-07-06

**Authors:** Leila M. Soravia, Ariane Orosz, Simon Schwab, Masahito Nakataki, Roland Wiest, Andrea Federspiel

**Affiliations:** ^1^ Division of Systems Neuroscience of PsychopathologyUniversity Hospital of PsychiatryUniversity of BernBolligenstrasse 1113000BernSwitzerland; ^2^ Department of PsychiatryUniversity of Tokushima3‐18‐15 Kuramoto‐choTokushima770‐8503Japan; ^3^ Department of Diagnostic and Interventional NeuroradiologyInselspitalUniversity Hospital of BernFreiburgstrasse 43010BernSwitzerland

**Keywords:** Anticipatory anxiety, arterial spin labeling, cerebral blood flow, cognitive‐behavioral therapy, fear processing, phobia

## Abstract

**Background:**

Imaging studies have provided evidence that cognitive‐behavioral therapy (CBT) is able to change brain activation in phobic patients in response to threatening stimuli. The changes occurred in both emotion‐generating and modulatory regions. In this study, we use a data‐driven approach to explore resting state cerebral blood flow (CBF) measured by arterial spin labeling (ASL), before and after CBT.

**Methods:**

Eight female patients with spider phobia were scanned before and 1 month after an exposure‐based group therapy for spider phobia. Each MRI session consisted of an ASL resting state measurement acquired before and after a symptom provocation task involving the showing of spider pictures in the scanner. The first ASL acquisition measured anticipatory anxiety and the second measured postprocessing of phobia‐relevant stimuli.

**Results:**

Cognitive‐behavioral therapy significantly reduced spider phobic symptoms in all patients. Symptom reduction during anticipatory anxiety was accompanied by reduced bilateral CBF in the parahippocampal gyrus, ventral anterior thalamus, Brodmann area 8, and the anterior cingulate cortex. During postprocessing of phobia‐relevant stimuli, patients showed reduced CBF in the bilateral insula, components of the motor cortex, and areas associated with language functions.

**Conclusions:**

Longitudinal CBF dynamics following CBT were in concordance with results from several studies using BOLD fMRI to investigate the effects of psychotherapy on brain activity. CBF can be quantified by ASL, with the principal advantage of sensitivity to slow variations in neural activity and task independence. Therefore, ASL may be a suitable method for monitoring and evaluating the efficacy of psychotherapy or pharmacotherapy approaches.

## Introduction

A specific phobia, such as spider phobia, is an anxiety disorder characterized by rapid, exaggerated, and persistent fear responses to a phobic situation or object, which interferes with functioning or causes significant distress (APA, 1994; American Psychiatric Association, 2013). Exposure to, or even the anticipation of the phobic stimulus, almost invariably provokes the retrieval of associated fear memories, leading to intense distress, anxiety, and/or avoidance of the feared object or situation (Foa and Kozak 1986; de Quervain and Margraf 2008; Duval et al. 2015). Phobic individuals tend to construct highly negative images of the phobic stimulus, which substantially contribute to anticipatory anxiety, as well as negative postevent processing (Laposa et al. 2014). Such images are usually associated with explicit fearful memories of past phobic experiences. The fearful memories then reinforce negative beliefs and strengthen the phobic response (Rapee and Heimberg 1997; Fehm and Margraf 2002). The anticipation and avoidance of potential future harm, and the retrieval of fearful memories, both seem to play a crucial role in the symptomatology and maintenance of specific phobias. There is extensive evidence that cognitive‐behavioral therapy (CBT), including exposure and cognitive restructuring, is the method of choice for the treatment of specific phobias (Hermans et al. 2006; McNally 2007; Bentz et al. 2010).

In recent decades, attempts have been made to identify the underlying neural activity of specific phobias. Imaging studies have shown hyperactivation in response to phobic stimuli in brain regions such as the amygdala, medial temporal lobe (MTL), insula, fusiform gyrus, and dorsal anterior cingulate cortex (ACC). These regions play a key role in identifying fearful stimuli and generating fear responses (Duval et al. 2015). Concurrent with these hyperactivations, decreased activity has been observed in the medial orbitofrontal cortex in response to phobic stimuli (Squire 1992; Moser and Moser 1998; Cabeza and Nyberg 2000; de Quervain et al. 2003; Schienle et al. 2007). This brain region is crucially involved in self‐regulation of emotions and learning (Schienle et al. 2007). The MTL, which consists of the hippocampus and parahippocampal gyrus, is involved in the retrieval of declarative and autobiographical memories in humans (Squire 1992; Moser and Moser 1998; Cabeza and Nyberg 2000; de Quervain et al. 2003). Thus, the MTL is of particular interest in the context of specific phobia, as memory processes, such as the retrieval of aversive memory, play a key role in maintenance of the disorder (de Quervain et al. 2009). Several PET and fMRI studies have shown that following successful psychotherapy or pharmacotherapy, visual associative cortical areas, including the insula, amygdala and MTL, are less activated during visual symptom provocation, while prefrontal cortical regions show increased activation (Furmark et al. 2002; Paquette et al. 2003; Sakamoto et al. 2005; Schienle et al. 2005, 2009; Straube et al. 2006).

Currently, several imaging studies have demonstrated that CBT is able to change brain activation in response to threatening stimuli in both emotion‐generating and modulatory regions (Duval et al. 2015). However, the assessment of neural correlates of therapeutic effects using conventional blood oxygenation level‐dependent (BOLD) fMRI remains challenging. Although BOLD enables resting state calculations, it is limited to a qualitative measure, and does not offer quantitative information on baseline brain metabolism. Therefore, the measurement of cerebral blood flow (CBF) seems a potential solution to overcome this putative limitation. CBF can be quantified by arterial spin labeling (ASL), a noninvasive functional MRI method that uses water molecules in arteries as an intrinsic tracer. The ASL method has a major advantage in that it is sensitive to slow variations in neural activity, and offers an absolute and direct measure of CBF.

The aim of this study was to explore whether resting CBF measures, taken before and after successful CBT, undergo changes comparable with those found using fMRI. In contrast to earlier studies that applied hypothesis‐driven seed‐based analyses (Klumpp et al. 2014), we performed a data‐driven approach using voxel‐wise calculations. To the best of our knowledge, no such voxel‐wise analyses have yet been performed. We were specifically interested in changes in brain activity during the conditions of anticipatory anxiety, as well as postprocessing of phobia‐relevant stimuli.

## Method

### Participants

Eight female subjects (mean age: 28.25, SD: 9.1 years; range: 20–46 years) who completed an exposure‐based cognitive behavioral group therapy (CBGT) for spider phobia were included in the analysis. They were selected from a subject group participating in a double‐blind placebo controlled study investigating the effect of cortisol on the outcome of an exposure‐based short‐term group therapy for spider phobia (Soravia et al. 2014). Diagnoses were based on the *Diagnostic and Statistical Manual of Mental Disorders*, fourth edition [DSM‐IV; (APA, 1994)], using a computer‐based structured clinical interview [DIA‐X; (Wittchen and Pfister 1997; Essau et al. 1999)], which was based on the Composite International Diagnostic Interview [CIDI; (Rubio‐Stipec and Bravo 1991)]. The German version (Rinck et al. 2002) of the Fear of Spider Questionnaire [FSQ; (Szymanski and O'Donohue 1995)] was used to further assess the severity of spider phobia. This study included subjects from the placebo group, i.e., subjects who received group therapy but no cortisol. The crucial inclusion criterion was an ASL signal of sufficient quality. Subjects were recruited via advertisements. Exclusion criteria included history of head injury, acute or chronic medical conditions, psychiatric disorders other than a specific phobia of spiders, psychotropic drug treatment, neurological diseases, current drug or alcohol abuse, or any contraindication to MRI.

Written informed consent was obtained from subjects after they received a complete description of the study. The study was approved by the ethics committee of the Canton of Bern, Switzerland (Nr. 161/07), in accordance with the principles of the Declaration of Helsinki (Rickham 1964).

### Experimental procedure

Patients were scanned before, and 1 month after the successful completion of an exposure‐based group therapy for spider phobia. Each MRI session consisted of two 6 min ASL resting state measurements, taken before and after a symptom provocation task, which involved the showing of spider pictures in the scanner. The picture set consisted of 80 phobic and nonphobic pictures, which were selected from the International Affective Picture System (Lang et al. 2008). The pictures were the same for both MRI sessions, although the order of presentation was random. Each image was presented for 5 sec and was followed by a fixation cross for 1 sec. Each block (picture, fixation) was followed by a randomly jittered interval of 3–5 sec before the next block commenced. The picture task lasted approximately 18 min. The first ASL measurement acquired before the picture demonstration was to represent a state of anticipatory anxiety, as the patients were previously instructed that they would be exposed to spider pictures while in the scanner. The second ASL session, acquired after the symptom provocation task, was intended to measure CBF during postevent processing of phobia‐relevant stimuli. The MRI experiments took place at the University Institute for Diagnostic and Interventional Neuroradiology, University Hospital of Bern, Switzerland, between 2 pm and 5 pm. Upon arrival, participants were informed about the experimental procedure and about the exposure to spider pictures while in the scanner. To assess treatment success, the questionnaire on spider phobic symptoms (FSQ) used during the diagnostic phase was again completed after session 2. Patients were asked to fill out the Spielberger State Anxiety Inventory [STAI‐SI; (Spielberger et al. 1970)] before and after each scanning session. Additionally, patients were asked to rate the experienced anxiety on a visual scale from 0 to 100, while looking at the pictures in the scanner.

### Image acquisition and ASL data analysis

Imaging was performed on a 3T Siemens Magnetom Trio TIM system (Siemens, Erlangen, Germany) equipped with a 12‐channel head coil. The pseudo continuous ASL parameters were: 13 slices, 6.5‐mm slice thickness, FOV = 230 × 230 mm^2^, matrix = 128 × 128, TR/TE/*τ*/PLD = 3500/18/1720/1100 msec, and FA = 25°. A balanced labeling technique (Wu et al. 2007; Dai et al. 2008) was used, with a mean slice‐selective gradient (Gz) of 0.6 mT/m. Fifty label/control pairs were acquired. In session 1, a 3D T1‐weighted structural scan was acquired using a modified driven equilibrium Fourier transform (mdeft) sequence. The parameters included 176 sagittal slices with 1.0‐mm thickness, voxel size = 1 × 1 × 1 mm^3^, TR/TE = 7.92/2.48 msec, FA = 16°, FOV = 256 × 256 mm^2^, matrix size = 256 × 256, Ti = 910 msec. These parameters were selected to achieve an optimal contrast‐to‐noise ratio (Johanson et al. 2006). Data were subjected to standard preprocessing steps using Statistical Parametric Mapping software (SPM8; Wellcome Trust Centre for Neuroimaging London; www.fil.ion.ucl.ac.uk/spm). In‐house Matlab routines (The MathWorks, Natick, MA) were used to calculate absolute CBF maps. ASL images were first motion corrected, and then CBF was quantified using a single‐compartment model (T1b 1650 msec, labeling efficiency 0.85, blood‐tissue partition coefficient 0.9 mL/g). CBF images were coregistered to the T1 images, normalized into standard MNI space, and smoothed with an 8‐mm full‐width at half maximum Gaussian kernel.

### Statistical procedures

Phobic symptoms were assessed at Session 1 and 2 using the FSQ (Rinck et al. 2002). Whole‐brain CBF was quantified using a gray matter mask created from the T1 images during the segmentation step. To compare the mean global CBF across the four ASL runs, values were submitted to a repeated measures analysis of variance (ANOVA) with ASL run as the within‐subject factor. To discretely explore anticipatory anxiety (prepicture task ASL runs) and postevent processing (postpicture task ASL runs), two separate voxel‐wise *T*‐tests were performed using a flexible factorial design with the factors subject (*N* = 8) and session (Session 1 vs. Session 2). This voxel‐wise analysis resulted in statistical maps. In these maps, we localized all significant clusters (cluster size threshold = 10 voxels) with a probability of <0.05. This procedure generated the regions of interest used to extract the mean regional CBF values. Clusters belonging to the same region were merged before CBF quantification. Regional CBF values were adjusted to the global CBF by dividing the individual regional CBF by the corresponding global CBF value.

Adjusted CBF data and FSQ scores were analyzed using SPSS 22 (SPSS 22, Statistical Package).

## Results

Cognitive behavioral group therapy led to a significant decrease in phobic symptoms in all patients, as measured by the FSQ (range, MR Session 1: 79–86; MR Session 2: 19–77; *T* = 3.3, df = 7, *P* = 0.013, *d* = 1.48). Participants did not differ in terms of state anxiety between MRI measurements 1 and 2, before and after the scanning sessions (*P* > 0.225), but they reported significantly less experienced anxiety while looking at the spider pictures after CBGT treatment (mean ± SD, MR session 1: 81.0 ± 11.37; MR session 2: 39.4 ± 22.0; *T* = 5.05, df = 7, *P* = 0.001, *d* = 2.34).

The ANOVA revealed no significant difference in global CBF across the four ASL runs (*F* = 2.37, df = 3, 21, *P* > 0.05), although there was a slight decline from Session 1 (prestimuli: 55.7 ± 9.2 mL/100 g/min, poststimuli: 54.5 ± 6.4 mL/100 g/min) to Session 2 (prestimuli: 50.7 ± 4.3 mL/100 g/min, poststimuli: 50.6 ± 4.0 mL/100 g/min). Therefore, state‐dependent global CBF differences can be excluded and the regional CBF changes are assumed to represent fear‐specific alterations in response to therapy.

**Figure 1:**
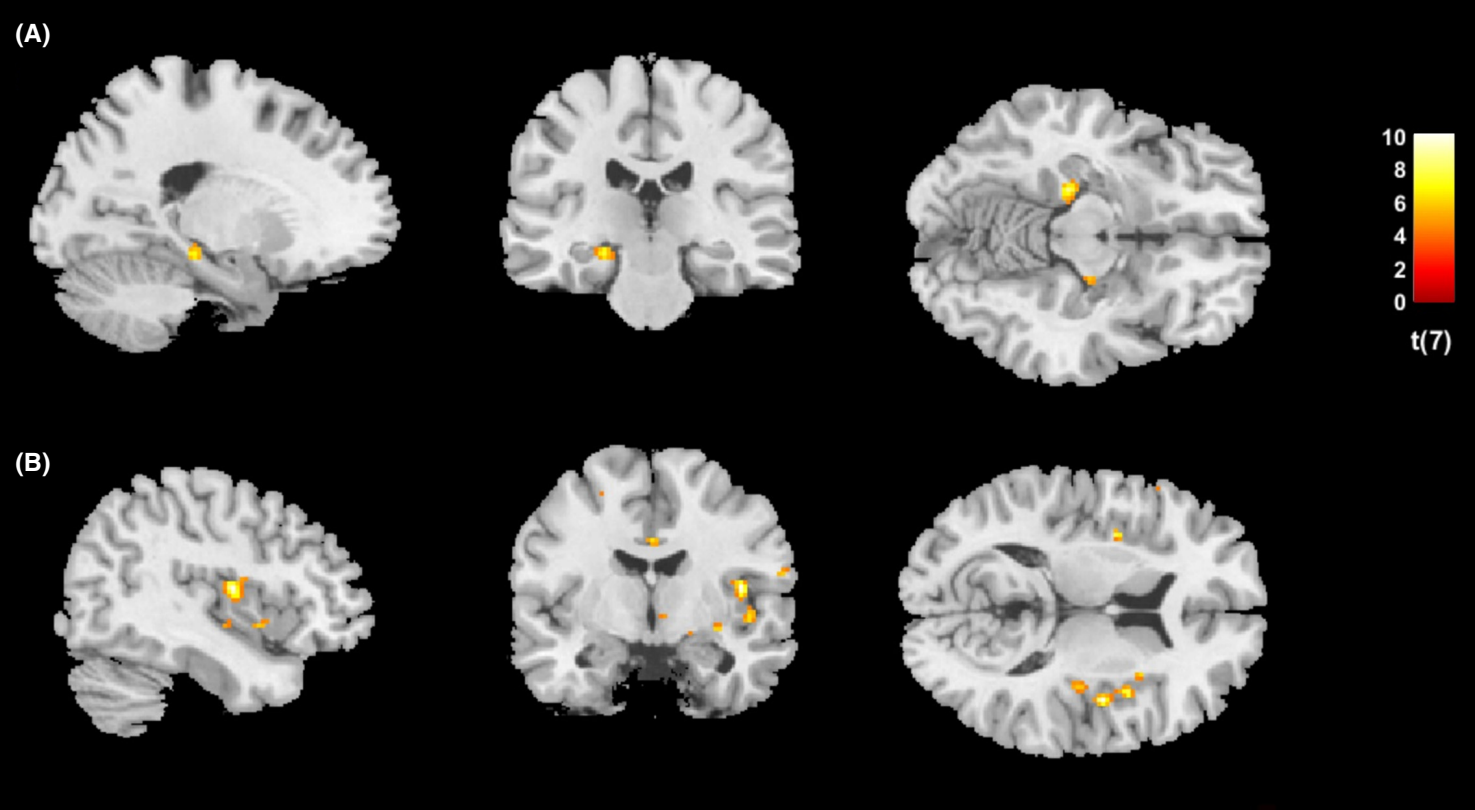
Effects of successful CBT on CBF during anticipatory fear and postevent processing of fear‐inducing stimuli (A) *T*‐test of the presymptom‐provocation task ASL acquisitions investigating anticipatory fear in Sessions 1 and 2 (before CBT vs. after CBT), showing predominantly the left and partial right parahippocampal gyrus, *z* = −12; *T* = 4.78, df = 7, *P* < 0.001, *d* = 1.60 (B) *T*‐test of the postsymptom‐provocation task ASL acquisitions investigating postevent processing of fear‐inducing stimuli in Sessions 1 and 2 (before CBT vs. after CBT), showing bilateral insula and part of the anterior cingulate cortex, with the right insula producing more significant clusters, *z* = 12; *T* = 4.78, df = 7, *P* < 0.001, *d* = 1.64. CBT, cognitive‐behavioral therapy; CBF, cerebral blood flow; ASL, arterial spin labeling.

Nonetheless, the following results were calculated using CBF values adjusted to the corresponding global CBF. From Session 1 to 2, CBF decreased in all clusters, indicating an effect of CBGT on both anticipatory fear and postevent processing of fearful stimuli (see Table 1A and B). After successful psychotherapy, patients showed a significant prestimuli anxiety‐related reduction in CBF activity in the ventral anterior nucleus of the thalamus, bilateral parahippocampal gyrus (Fig. 1A), ventral ACC, and a part of the frontal cortex in Brodmann area (BA) 8. However, following the successful completion of psychotherapy, patients showed a CBF decline in the left and right insula (Fig. 1B), left thalamus, primary motor cortex (BA 6), premotor cortex (BA 4), and frontal cortex (BA10, BA7, BA22, and BA45), during postevent processing of fearful stimuli.

**Table 1:** (A) Anticipatory fear processing in anticipation of the presentation of spider pictures: ASL presymptom provocation task Session 1 versus ASL presymptom provocation task Session 2. (B) Fear post event processing after the presentation of spider pictures: ASL postsymptom provocation task Session 1 versus ASL postsymptom provocation task Session 2. The first three columns describe the clusters resulting from the *t*‐contrast comparing mean CBF images. The last three columns show the quantified CBF values and the result of the *T*‐test comparing the mean CBF values

Region	Cluster size	Coordinates			*T* peak level	CBF pre [mL/100 g/min]	CBF post [mL/100 g/min]	*T*‐test pre versus post (gCBF corrected)		
		*x*	*y*	*z*				*T*	*P*	*d*
(A)										
Thalamus ventral anterior nucleus	71	−2	−16	16	9.61	61.3 ± 11.9	44.0 ± 8.2	3.88	0.006	1.47
Parahippocampal left	45	−22	−26	−12	9.06	72.1 ± 7.2	50.2 ± 11.5	5.69	0.001	1.60
Parahippocampal right	11	20	−16	−12	5.94	64.3 ± 9.2	47.0 ± 11.7	3.01	0.020	1.08
Superior frontal gyrus left	13	−14	22	54	6.18	64.7 ± 8.3	49.8 ± 6.8	2.13	0.071	1.06
BA7 left	56	−42	−72	44	10.59	61.4 ± 6.9	50.9 ± 6.1	1.75	0.123	1.01
BA8 right	10	26	22	54	6.33	65.4 ± 9.2	53.1 ± 4.7	3.8	0.007	1.64
BA24/vACC right	a) 11	14	14	34	6.99					
	b) 21	12	6	42	6.65	46.6 ± 10.0	34.7 ± 8.2	2.8	0.026	0.81
BA39 left	a) 11	−44	−62	22	5.82					
	b) 15	−54	−66	22	5.3	72.7 ± 11.1	61.6 ± 11.1	2.17	0.067	0.38
BA40 right	10	46	−56	32	5.43	74.1 ± 7.5	60.5 ± 6.3	2.2	0.064	0.95
(B)										
Insula right	a) 315	42	−6	12	11.15	70.3 ± 6.2	54.5 ± 6.7	3.8	0.007	1.64
	b) 32	36	−18	10	7.26					
Insula left BA13	a) 22	−36	0	12	8.28	66.7 ± 7.8	53.9 ± 9.3	3.4	0.010	0.91
	b) 40	−36	22	8	8.06					
Thalamus left	16	−6	−12	0	8.33	70.1 ± 8.8	57.4 ± 9.3	2.9	0.021	1.13
BA4 right	17	62	−4	18	7.73	63.7 ± 6.6	53.0 ± 6.1	3.1	0.017	0.85
BA6 left/Premotor area	a) 54	−24	0	56	8.19	66.4 ± 9.1	49. 5 ± 6.1	4.9	0.002	1.89
	b) 31	−26	−10	52	7.13					
BA7 right	15	26	−76	48	9.25	46.2 ± 9.0	37.8 ± 8.3	3.3	0.014	0.64
BA9 right	13	6	40	20	7.06	79.0 ± 8.4	69.4 ± 8.4	1.8	0.109	0.67
BA10 left	42	−18	62	20	10.65	65.1 ± 8.1	53.8 ± 8.8	3.1	0.017	0.91
BA19*	38	−44	−82	26	7.57	52.1 ± 7.3	43.2 ± 8.7	2.3	0.052	0.68
BA22 left	13	−50	0	4	5.59	75.5 ± 6.0	60.0 ± 9.5	3.3	0.012	1.28
BA24/vACC	12	0	−6	34	6.98	72.4 ± 8.6	62.1 ± 9.3	1.7	0.137	0.84
BA40 left	17	−60	−18	18	7.36	64.0 ± 6.1	53.2 ± 7.4	0.06	0.953	0.02
BA43 right	17	66	−16	20	7.26	64.1 ± 8.1	53.5 ± 6.0	2.1	0.072	0.86
BA45 left/Broca's area	12	−58	20	8	6.10	57.3 ± 8.2	42.6 ± 6.7	6.9	0.000	1.03

ASL, arterial spin labeling; BA, Brodmann area; CBF, cerebral blood flow; *d*, Cohens' *d*, effect size; a) and b) refer to independent clusters within the same region; BA19*, left associated visual cortex; vACC, ventral anterior cingulate cortex.

## Discussion

The aim of this study was to use ASL to investigate whether CBT for spider phobia could modulate resting CBF in relation to conditions of anticipatory anxiety and postevent processing of phobia‐relevant stimuli. CBT significantly reduced spider phobic symptoms in all patients. After successful CBT for spider phobia, patients in the anticipatory anxiety state prior to the fear‐inducing stimuli, showed reduced CBF in the bilateral parahippocampal gyrus, ventral anterior thalamus, frontal eye fields (BA 8), and ACC. During postevent processing of phobia‐relevant stimuli, patients showed reduced CBF in the bilateral insula, components of the motor cortex (BA 4, 6), and areas associated with language functions (BA 7, 22, 45).

The changes in resting state CBF following successful CBT are in line with results from several studies investigating the effects of psychotherapy on brain activity using BOLD fMRI. According to a review on functional neuroimaging in specific phobia, most studies have shown abnormal activation in brain regions involved in emotion‐generation and emotion‐regulation, particularly the amygdala, ACC, thalamus, and insula. Activations are also reported for the prefrontal, orbitofrontal, and visual cortices, indicating impaired emotional modulation after exposure to phobic stimuli (Del Casale et al. 2012). From fMRI studies, it may be inferred that the MTL, which consists of the hippocampus and parahippocampal gyrus, is crucially involved in the retrieval of declarative and autobiographical memory in humans, and spatial and contextual memory in animals (Squire 1992; Moser and Moser 1998; Cabeza and Nyberg 2000; de Quervain et al. 2003). In the context of phobias, the MTL is of interest, as memory processes such as the retrieval of aversive memory play a key role in the maintenance of the disorder (de Quervain et al. 2009). A PET study in patients with social phobia reported that, following successful psychotherapy or pharmacotherapy, the MTL was less activated during a public speaking task (Furmark et al. 2002). Comparable results were reported by Paquette et al. (2003), who investigated the effects of CBT in patients with spider phobia. Patients showed high activation in the parahippocampal gyrus and the visual associative cortical areas during viewing of phobogenic stimuli before therapy. The visual areas may reflect a typical state of hypervigilance, while the parahippocampal activation may be related to an automatic reactivation of the contextual fear memory that led to the development of avoidance behavior and maintenance of the disorder (Paquette et al. 2003). However, following successful completion of therapy, significant activation was not found in the visual associative cortical areas, parahippocampal gyrus, or the dorsolateral prefrontal cortex. This further indicates the crucial role of the MTL in the fear circuit, and fear response.

A recent fMRI study by Münsterkötter et al. (2015) investigated the neuronal networks involved in sustained anticipatory anxiety toward an unpredictable threat in spider phobic patients. The findings indicated that the right ACC, insula, and the bed nucleus of the stria terminalis, all play a key role in the processing of anticipatory anxiety, supporting previous fMRI findings on anticipatory anxiety (Vuilleumier et al. 2001; Simmons et al. 2006; Straube et al. 2007; Wendt et al. 2008; Münsterkötter et al. 2015). Our finding of reduced CBF in the ACC during anticipatory anxiety following successful CBT, is therefore in line with their fMRI results. However, unlike Münsterkötter et al. (2015) or Straube et al. (2007), we did not find reduced CBF in the insula and the bed nucleus of the stria terminalis during anticipatory anxiety after successful psychotherapy. This may be due to our use of a pre‐ and posttreatment design within a patient group, as opposed to comparisons between patients and healthy control subjects, as used in the fMRI studies. Instead, after successful therapy, we found reduced CBF in the bilateral insula during postevent processing of phobic stimuli. According to Brozovich and Heimberg (2013), the two self‐focused thought processes of anticipatory anxiety and postevent processing are often intertwined, and occur at different times before and after phobic situations. Not only does the ACC appear to play an important role in the anticipation of fear, but so also does region BA 8, which is involved in frontal eye‐field control of visual attention. fMRI studies have shown that activation in BA 8 occurs when subjects experience uncertainty (Volz et al. 2003, 2004; Suzuki et al. 2015), which is comparable to the lasting state of anxious apprehension that occurs while anticipating an unpredictable threat, and is similar to the situation in this study with spider‐phobic patients. Hence, the reduced anticipatory anxiety achieved through CBT is associated with reduced CBF in this brain region.

Although the finding of reduced CBF in the bilateral insula following successful CBT is in accordance with several fMRI studies (Schienle et al. 2007), the finding of reduced CBF in components of the motor cortex and areas associated with language functions is not obvious. The motor cortex appears to play a role in the direct control of behavior and the planning of movement eliciting defensive behavior and action preparedness in response to negative emotional stimuli (Blakemore et al. 2016). An fMRI study investigating the effects of CBT on neuronal networks demonstrated the involvement of the inferior frontal gyrus (BA 45) in the pathology of panic disorders, and in the successful psychotherapy of such disorders (Kircher et al. 2013). Thus, we speculate that parts of the language system are involved in the cognitive processing occurring during symptom improvement in patients with panic disorders. Against expectations, we did not find any CBF changes in the orbitofrontal cortex, unlike other imaging studies that investigated the effects of CBT in different anxiety disorders (Johanson et al. 2006; Schienle et al. 2007).

Some limitations should be taken into consideration. The sample size was rather small and included only female patients with spider phobia, which makes the generalization of the results difficult. Specific measurements for anticipatory anxiety and postevent processing are missing from this study; these may have given more clinical information, which could have been directly linked to the CBF data. As the two experimental MRI measurements taken before and after CBT were identical, we cannot exclude the possibility of habituation effects, which may also account for the changes in CBF. The main limitation of this study is the lack of a control group. Although all patients showed a significant reduction in spider‐phobic symptoms after successful CBT, we cannot fully attribute the observed changes in brain response to the treatment. With the present design, we are not able to control for other factors that may be responsible for the observed changes. These could include habituation to the experimental procedure, repeated stimulus presentation, or memory retrieval effects. Schienle et al. (2007) used fMRI to investigate the effects of exposure therapy and symptom provocation in spider‐phobic patients. Patients in the treatment group showed a significant reduction in spider‐phobic symptoms and reduced activation of the insula following successful exposure therapy. However, patients in the control group showed no changes in spider‐phobic symptoms, but showed a reduction in the activation of the bilateral parahippocampal gyrus, and right and left medial orbitofrontal cortex (OFC). Contrasting the responses between the two groups, the treatment group showed greater activation in the bilateral medial OFC. Thus, we cannot exclude the possibility that the reduced CBF in the bilateral parahippocampal gyrus during anticipatory anxiety may have come from an uncontrolled habituation effect. Despite this, during postprocessing of phobia‐relevant stimuli, patients showed reduced CBF in the bilateral insula, which is in accordance with the results of Schienle et al. (2007). Nevertheless, it is important to note that changes in brain response may occur in the absence of clinical changes.

Compared to conventional BOLD fMRI, ASL enables the quantification of CBF and offers the major advantage of being sensitive to slow variations in neural activity (Wang et al. 2003). ASL during the resting state is capable of detecting subtle changes in regional CBF within nodes of specific anxiety‐related networks; these may be modulated by CBT. However, a recent ASL study found no changes in resting CBF after 4 days of meditation training, although anxiety symptoms were significantly reduced (Zeidan et al. 2014). Thus, this is the first ASL study to replicate results from different fMRI studies, and show that successful CBT for a phobia can change brain activity in the MTL and insula during the resting state. ASL also enables absolute quantification of CBF changes in the corresponding phobia‐associated regions. Therefore, ASL may be a suitable method for monitoring and evaluating the efficacy of psychotherapy or pharmacotherapy approaches.

## Conflict of Interest

The authors report no financial interests or potential conflicts of interest.
